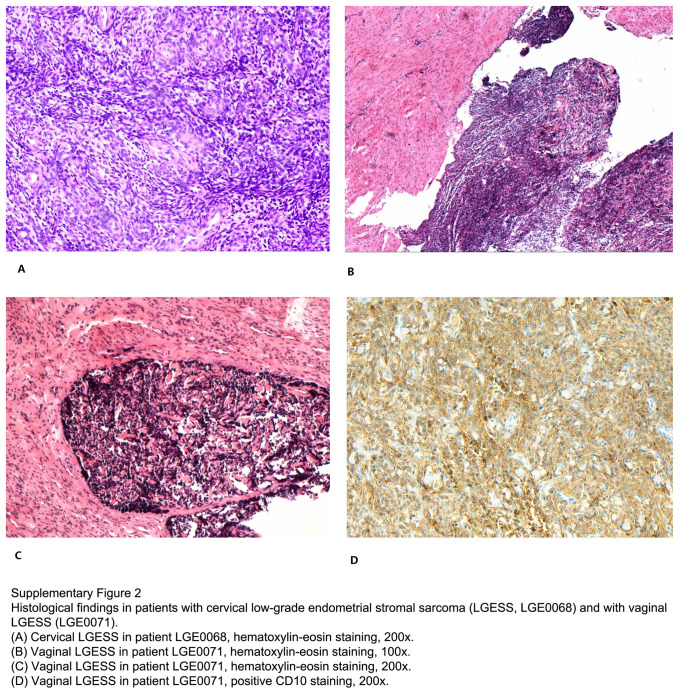# Prognosis of uterine and extrauterine low-grade endometrial stromal sarcoma: an observational cohort study

**DOI:** 10.1097/JS9.0000000000001146

**Published:** 2024-02-07

**Authors:** Qianwen Dai, Baolin Xu, Huanwen Wu, Yan You, Lei Li

**Affiliations:** a Department of Obstetrics and Gynecology; b State Key Laboratory for Complex, Severe and Rare Diseases; c Department of Pathology, Peking Union Medical College Hospital; d National Clinical Research Center for Obstetric and Gynecologic Diseases, Beijing; e Department of Obstetrics and Gynecology, the Second People’s Hospital of Jingdezhen, Jingdezhen, People’s Republic of China

**Keywords:** low-grade endometrial stromal sarcoma, ovarian preservation, recurrence, survival

## Abstract

**Objective:**

Little is known about the survival differences between uterine and extrauterine low-grade endometrial stromal sarcoma (LGESS). Survival outcomes, consisting of disease-free survivals and overall survivals (OS), were compared in these two entities.

**Methods:**

From February 2012 to June 2019, all primary LGESS cases and LGESS cases with first recurrence in the study center were reviewed. The clinicopathological characteristics and survival outcomes of extrauterine and uterine LGESS patients were compared for both primary and recurrent diseases.

**Results:**

During the study period, 143 patients with primary LGESS and 56 patients with recurrent LGESS were included and followed up to 1 June 2020, among whom 8 (5.6%) and 10 (17.8%) patients were identified as having extrauterine LGESS. Patients with primary and recurrent extrauterine LGESS had similar clinicopathological characteristics to those of patients with uterine LGESS. In primary or in recurrent LGESS cases, in univariate analysis, patients with uterine and extrauterine LGESS had similar disease-free intervals after the last treatment, and they also had similar OSs after the diagnosis. Ovarian preservation led to significantly increased recurrence for primary LGESS [hazard ratio (HR) 4.9, 95% CI: 2.3–10.1, *P*<0.001) and repeated recurrence for recurrent LGESS (HR 3.1, 95% CI: 1.3–7.3, *P*=0.009). Surgical treatment for recurrent LGESS decreased repeated recurrence after the first recurrence (HR 0.2, 95% CI: 0.1–0.7, *P*=0.006). No factors were found to be associated with the OS of primary or recurrent LGESS.

**Conclusion:**

The clinical characteristics and survival outcomes of extrauterine LGESS are similar to those of uterine LGESS. Surgery is the treatment of choice for recurrent LGESS. Ovarian preservation is detrimental to disease-free survival but not to OS in both uterine and extrauterine LGESS.

## Introduction

Highlights

- Extrauterine low-grade endometrial stromal sarcoma (LGESS) had similar clinical characteristics and survival outcomes compared with uterine LGESS in primary or in recurrent disease status.
- Surgical treatment is the choice for all sorts of LGESS to achieve advanced disease-free survivals.
- Ovarian preservation would cause detriment to disease-free survival but not to overall survival.
- No patient with extrauterine LGESS achieved successful conception.

Low-grade endometrial stromal sarcoma (LGESS) is a malignant tumor composed of cells resembling stromal cells of the proliferative-phase endometrium, displaying permeative, infiltrative growth into the myometrium and/or Iymphovascular spaces^1^. LGESS represents <1% of all uterine malignancies, accounting for only 0.2% of female genital tract malignancies, but is the second most common uterine malignant mesenchymal tumor^1^. LGESS had a favorable prognosis^2^, with a 5-year disease-specific survival rate of 90% for stage I or II disease and of 50% for stage III or IV disease^3,4^. Although less common, primary extrauterine LGESS, or low-grade endometrioid stroma sarcoma, namely, LGESS without uterus involved, has been studied in many case reports. However, the prognostic significance of extrauterine LGESS is not as well-known as that of uterine LGESS due to the scarcity of cases for study. Some authors have suggested that extrauterine LGESS may have a similar survival prognosis to its uterine counterpart^5^, but others suggest that it tends to have a worse prognosis^6^. Ovarian LGESS seems to have a much better prognosis than other primary ovarian sarcomas^7^. There is no direct evidence demonstrating the prognostic differences between uterine and extrauterine LGESS. Recent studies either had very limited case samples^8,9^ or simply described the extrauterine LGESS cases themselves^5,7^. These factors have limited an in-depth understanding of the oncological behavior of extrauterine LGESS.

In this observational cohort study, all patients diagnosed with LGESS from February 2012 to June 2019 in our study center and were reviewed and classified into the primary and recurrent LGESS cohorts. The clinical characteristics, treatment characteristics, and prognoses of extrauterine LGESS were compared to those of uterine LGESS as to characterize the specific survival outcomes of extrauterine LGESS.

## Methods

### Ethical approval

The Institutional Review Board from the study center has approved this observational study (No. SK-1289). All procedures performed in the study involving human participants were in accordance with the ethical standards of the institutional and National Research Committee and with the 1964 Declaration of Helsinki and its later amendments or comparable ethical standards. This work has been reported in accordance with STROCSS standards^10^.

### Study design and participants

This is an observational cohort study. Patients diagnosed and treated for LGESS in one study center, from February 2012 to June 2019 were retrospectively included. LGESS is defined as a malignant stromal tumor with cells resembling proliferative-phase endometrial stroma and displaying infiltrative (permeative) growth with or without lymphovascular invasion^1^. Primary extrauterine LGESS is defined as a low-grade mesenchymal neoplasm with a morphology resembling that of proliferative-type endometrial stroma without uterus involved^1^.

The main study outcomes were survival outcomes, and the secondary outcomes were fertility outcomes and factors relevant to survival and fertility outcomes. Follow-up of fertility and oncologic outcomes were carried out up to 1 June 2020. The prognosis of extrauterine LGESS was compared with that of primary uterine LGESS during the same periods; the prognosis of recurrent extrauterine LGESS was also compared with that of recurrent uterine LGESS in terms of survival outcomes after first recurrence. For primary LGESS cohort, only patients undergoing surgical treatment during the study period were included. For recurrent LGESS, all patients were included no matter whether they underwent surgical treatment or not. Patients with pathological findings rather than LGESS, even patients with mixed high-grade and low-grade endometrial stromal sarcoma, were excluded. As some patients developed recurrence during the study period, their survival outcomes were also included in the analysis for patients after first recurrence.

All clinicopathological data were collected from the case reports. Data on personal medical history and survival outcomes were collected by outpatient interviews. All histological findings of LGESS were strictly reviewed and confirmed by two pathologists (Y.Y. and H.W.). For extrauterine LGESS, close attention was paid to the examination of uterine histology. Efforts were made to ascertain the status of the uterus if this information was not obvious at the time the extrauterine foci were removed. If the patient had her uterus removed before diagnosis of extrauterine LGESS, the previous uterine pathology, if applicable, was comprehensively reviewed. If the uterus remained intact at first treatment, the uterine pathology after surgery for recurrence was checked. If uterine histology was not available, indirect evidences of uterine lesions, that is, histology from endometrial sampling and imaging evaluation, were reviewed together.

### Interventions and measures

Data on surgical treatment, including surgical routes, retroperitoneal lymphadenectomy, and pathological results (FIGO staging, lymphovascular space invasion, metastasis to lymph nodes) were collected from the case and pathology reports. For recurrent LGESS disease, surgical routes and residual lesions reported by surgical records were also collected from the case reports. Attention was given to the chemotherapy regimens and courses, hormone therapy regimens and courses, and radiotherapy. Oncologic outcomes consisted of disease-free survival (DFS) since last treatment and overall survival (OS) since the diagnosis, which were followed up to 1 June 2020. Fertility outcomes consisted of conception rates and birth rates during the same follow-up periods.

In this study, ovarian preservation denotes preservation of at least one ovary with or without an intact uterus. All recurrences were confirmed by histological diagnosis by biopsy and/or repeated surgeries. All deaths were confirmed by certification of death.

### Statistics

Comparisons of continuous variables were conducted with parametric methods if assumptions of normal distribution were confirmed. Non-normally distributed variables and categorical data were compared between various groups of specific clinicopathological characteristics by using nonparametric tests. Survival curves were generated with the Kaplan–Meier method, and proportional hazards models were used to estimate the hazard ratios (HRs), 95% CI and *P* values for the effects of treatment modalities on DFS, PFI, and OS. Unless otherwise stated, all analyses were performed with a two-sided significance level (*P* values) of 0.05 and were conducted with the use of the software Statistical Product and Service Solutions (SPSS) Statistics 20.0 (IBM Corporation).

## Results

### Patient characteristics

**Figure 1:**
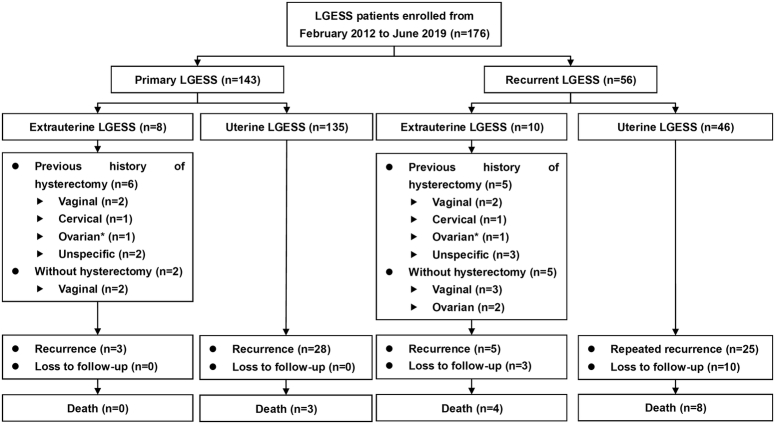
Flow diagram of the study. LGESS, low-grade endometrial stromal sarcoma. * In our study, ovarian low-grade endometrioid stroma sarcoma was regarded as LGESS.

The flow diagram is presented in Figure 1. During the study periods, among 143 patients with primary disease and 56 patients with recurrent disease were enrolled, 8 (5.6%) and 10 patients (17.8%) were identified as having extrauterine LGESS, respectively. The total number of patients with extrauterine LGESS was 15, since three patients with primary diseases experienced recurrences during the study period. The baseline characteristics of the patients with primary and recurrent disease are summarized in Table 1 and Table 2, respectively. The patients with extrauterine and uterine LGESS were well balanced in terms of almost all these characteristics for both primary disease and recurrent disease. However, due to the location of the disease, 10 and 4 patients with uterine and extrauterine LGESS underwent hysteroscopy and vaginal surgeries, respectively, which was unique in this population. All patients’ data were supplied in Supplementary Table 1 (Supplemental Digital Content 1, http://links.lww.com/JS9/B820 and Supplementary Table 2, Supplemental Digital Content 2, http://links.lww.com/JS9/B821).

**Table 1:** Epidemiological, surgical, and pathological characteristics between patients with primary LGESS.

	Uterine LGESS (*n*=135)	Primary extrauterine LGESS (*n*=8)	*P*
Age at diagnosis (years), median (range)	47 (30–45)	43 (15–41)	0.261
Gravidity, median (range)	3 (0–3)	2 (0–2)	0.312
Parity, median (range)	2 (0–2)	1 (0–1)	0.089
Fertility sparing, *n* (%)	21 (15.6)	2 (25.0)	0.378
Live birth, n/n (%)	7/21 (33.3)	0/2	0.474
Ovarian preservation, *n* (%)	42 (31.1)	2 (25.0)	0.531
Surgical routes, *n* (%)			<0.001
Hysteroscopy	10 (7.4)	0 (0.0)	
Laparoscopy	63 (46.7)	0 (0.0)	
Laparotomy	62 (45.9)	4 (50.0)	
Vaginal surgery	0 (0.0)	4 (50.0)	
Retroperitoneal lymphadenectomy, *n* (%)	18 (13.3)	0 (0.0)	0.331
Max diameter of the tumor (mm), median (range)	59 (29–70)	60 (13–66)	0.876
FIGO stages			0.156
Stage I–II	124 (91.1)	6 (75.0)	
Stage III–IV	11 (8.9)	2 (25.0)	
LVSI, n/n (%)	25/120 (20.8)	0/7 (0.0)	0.207
Positive estrogen receptor expression, n/n (%)	123/135 (91.1)	8/8 (100.0)	0.487
Positive progestrone receptor expression, n/n (%)	116/135 (85.9)	7/8 (87.5)	0.690
Mitotic activities >5 per high-powered field, n/n (%)	30/135 (22.2)	1/8 (12.5)	0.450
Metastasis to retroperitoneal lymph nodes, n/n (%)	61 (45.2)	4 (50.0)	0.535
Hormone treatment, *n* (%)			
Hormone treatment period (months), median (range)	18 (3–19)	9 (2–12)	0.636
Chemotherapy, *n* (%)	10 (7.4)	2 (25.0)	0.136
Radiotherapy, *n* (%)	15 (11.1)	2 (25.0)	0.243
Recurrence, *n* (%)	28 (20.7)	3 (37.5)	0.237
Disease-free survival (months), median (range)	51 (24–55)	39 (5–44)	0.236
Mortality, *n* (%)	3 (2.2)	0 (0.0)	0.840
Overall survival (months), median (range)	64 (24–67)	48 (10–51)	0.109

LGESS, low-grade endometrial stromal sarcoma; LVSI, lymphovascular space invasion.

**Table 2:** Epidemiological, surgical, and pathological characteristics between patients with recurrent LGESS.

	Uterine LGESS (*n*=46)	Primary extrauterine LGESS (*n*=10)	*P*
LVSI, n/n (%)	6/28 (21.4)	0/3 (0.0)	0.512
Positive estrogen receptor expression, n/n (%)	42/46 (91.3)	9/10 (90.0)	0.641
Positive progestrone receptor expression, n/n (%)	40/46 (87.0)	10/10 (100.0)	0.288
Mitotic activities >5 per high-powered field, n/n (%)	7/46 (15.2)	4 /10 (40.0)	0.093
DFS after first treatment (months), median (range)	31 (5-188)	78 (18-133)	0.167
Ages at diagnosis of recurrence (years), median (range)	40 (17–70)	53 (36–61)	0.063
Extrapelvic recurrence, *n* (%)	20 (43.5)	3 (30.0)	0.339
Symptomatic recurrence, *n* (%)	17 (37.0)	6 (60.0)	0.162
Repeated surgeries for first recurrence, *n* (%)	42 (91.3)	8 (80.0)	0.289
With residual lesions, n/n (%)	3/42 (7.1)	0/8 (0.0)	0.586
Chemotherapy for recurrence, *n* (%)	14 (30.4)	3 (30.0)	0.648
Radiotherapy for recurrence, *n* (%)	15 (32.6)	3 (30.0)	0.595
Hormone treatment for recurrence, *n* (%)	21 (45.7)	5 (50.0)	0.537
Fertility sparing			
Fertility sparing after first treatment, *n* (%)	13 (28.3)	4 (40.0)	0.352
Fertility sparing after first recurrence, *n* (%)	4/13 (30.8)	1/3 (33.3)	0.705
Ovarian preservation			
Ovarian preservation after first treatment, *n* (%)	29 (63.0)	6 (60.0)	0.563
Ovarian preservation after first recurrence, *n* (%)	5/27 (18.5)	1/5 (20.0)	0.673
Loss to follow-up, *n* (%)	10 (21.7)	3 (30.0)	0.423
Repeated recurrence, n/n (%)	24/36 (66.7)	6/7 (85.7)	0.303
PFI^a^ (months), median (range)	17 (1–101)	39 (8–121)	0.177
Death, n/n (%)	8/36 (22.2)	4/7 (57.1)	0.081
OS^b^ (months), median (range)	44 (3–349)	42 (21–229)	0.961

a PFI denotes progression-free interval since last treatment for first recurrence.

b OS denotes overall survival since the diagnosis of first recurrence.

DFS, disease-free survival; LGESS, low-grade endometrial stromal sarcoma; OS, overall survival; PFI, progression-free interval.

The clinicopathological characteristics of all 15 patients with extrauterine LGESS are listed in Table 3. No patient underwent myomectomy previously, by either laparoscopic or open surgery. In 10 cases, the uterus removed before the diagnosis of LGESS due to adenomyosis (two cases) or leiomyoma (eight cases). The median interval from hysterectomy to the diagnosis of LGESS was 93.5 (range 39–148) months. Two examples of vaginal LGESS were illustrated in Supplementary Figure 1 (Supplemental Digital Content 3, http://links.lww.com/JS9/B822). Among 10 patients with a hysterectomy history, 6 had a comprehensive review of the uterine histology, which had ruled out occult LGESS. Among five patients with an intact uterus at first treatment, one was proved of normal histology confirmed by pathological examination in specimens of recurrence, one patient underwent endometrial sampling at first treatment, and the pathological examination was confirmed normal; the other three had no available histology for review, and their extrauterine LGESS were diagnosed by surgical records, pathological reports, and imaging reviews. In total, 8 of 15 patients had direct evidence of extrauterine LGESS. Among seven patients without direct evidences of extrauterine LGESS were all in the recurrent cohort (LGE0008, LGE0010, LGE0013, LGE0014, LGE0016, LGE0018, and LGE0048).

**Table 3:** Detailed epidemiological characteristics, treatment and prognosis of patients with extrauterine LGESS.

No	Age at diagnosis (years)	Gravidity / parity	LGESS sites	EM	Hysterectomy routes	Uterine pathology	Interval from hysterectomy to diagnosis (months)	Tumor diameter (mm)	Hormone treatment	ChemoX	RadioX	Recurrent sites	DFS (months)	OS (months)
LGE0008	35	1/0	Ovarian	Yes	N/A	N/A	N/A	250	GnRHa 3 months	None	No	Pelvic cavity	39	96
LGE0010	41	3/1	Vaginal	No	N/A	N/A	N/A	30	None	None	No	Uterus, ovaries	95	158
LGE0011	36	3/2	Vaginal	No	N/A	Normal^a^	N/A	30	None	None	No	Vagina	35	69
LGE0012	48	3/1	Vaginal	No	N/A	N/A^b^	N/A	40	None	None	No	Vagina	47	87
LGE0013	46	2/1	Ovarian	No	Open	Leiomyoma^c^	110	45	None	None	No	Ovaries	26	122
LGE0014	68	5/3	Ovarian	No	Open	Leiomyoma^c^	63	170	None	None	No	Pelvic cavity	96	322
LGE0016	56	0/0	Ovarian	No	Open subtotal	Leiomyoma^c^	60	120	Progestin 12 months	PEI * 3 courses	Yes	Pelvic cavity	133	174
LGE0017	50	3/1	Unspecific	Yes	Open	Leiomyoma^d^	97	147	Aromatase inhibitor 30 months	None	No	Peritoneal and pelvic cavity, liver, retroperitoneal lymph nodes	78	98
LGE0018	63	1/1	Vaginal	No	Open	Leiomyoma^c^	60	40	None	None	Yes	Pelvic cavity	122	256
LGE0048	62	2/2	Ovarian	No	N/A	N/A	N/A	50	None	PEI * 6 courses	No	Pelvic cavity	18	54
LGE0068	53	3/3	Cervical	Yes	Open subtotal	Leiomyoma^d^	148	29	Progestin 30 months	None	Yes	Disease-free	42	42
LGE0069	30	0/0	Ovarian	Yes	Open	Adenomyosis^d^	90	103	Progestin 3 months	PEI * 3 courses	No	Disease-free	58	58
LGE0070	61	5/2	Unspecific	No	Open	Leiomyoma^d^	148	102	None	PEI * 5 courses	No	Disease-free	101	101
LGE0071	36	2/2	Vaginal	Yes	Open	Adenomyosis^d^	97	78	Progestin 6 months	None	Yes	Disease-free	24	24
LGE0072	45	2/1	Vaginal	Yes	Laparoscopic	Leiomyoma^d^	39	35	None	None	No	Disease-free	54	54

a The normal histology was confirmed by pathological examination in specimen of recurrence.

b This patient underwent endometrial sampling at first treatment, and the pathological examination was confirmed normal.

c These histological results had not been reviewed and confirmed due to lack of available materials.

d These histological results had been reviewed and confirmed.

ChemoX, chemotherapy; DFS, disease-free survival; EM, endometriosis; GnRHa, gonadotropin-releasing hormone agonist; LGESS, low-grade endometrial stromal sarcoma; N/A, not available; OS, overall survival; PEI, chemotherapy with cisplatin, etoposide and ifosfamide; RadioX, radiotherapy.

Of the 15 patients with extrauterine LGESS, 6 had ovarian LGESS, 6 had vaginal LGESS, 1 had cervical LGESS after subtotal hysterectomy, and 2 had nonspecific disease origins since they had multiple loci in the pelvic cavity. Endometriosis was found in 6 (40.0%) patients (Table 3). The histological findings in patients with cervical LGESS (LGE0068) and with vaginal LGESS (LGE0071) were illustrated in Supplementary Figure 2 (Supplemental Digital Content 4, http://links.lww.com/JS9/B823).

### Survival outcomes of primary LGESS

In patients with primary uterine and extrauterine LGESS, 28 of 135 (20.7%) and 3 of 8 (37.5%) developed recurrence, and 3 (2.2%) and 0 (0.0%) died, respectively. The median DFS and OS of primary LGESS patients were 39.3 months (range 5–101) and 52.9 (10–101) months, respectively.

### Survival difference between uterine and extrauterine LGESS in primary cases

**Figure 2:**
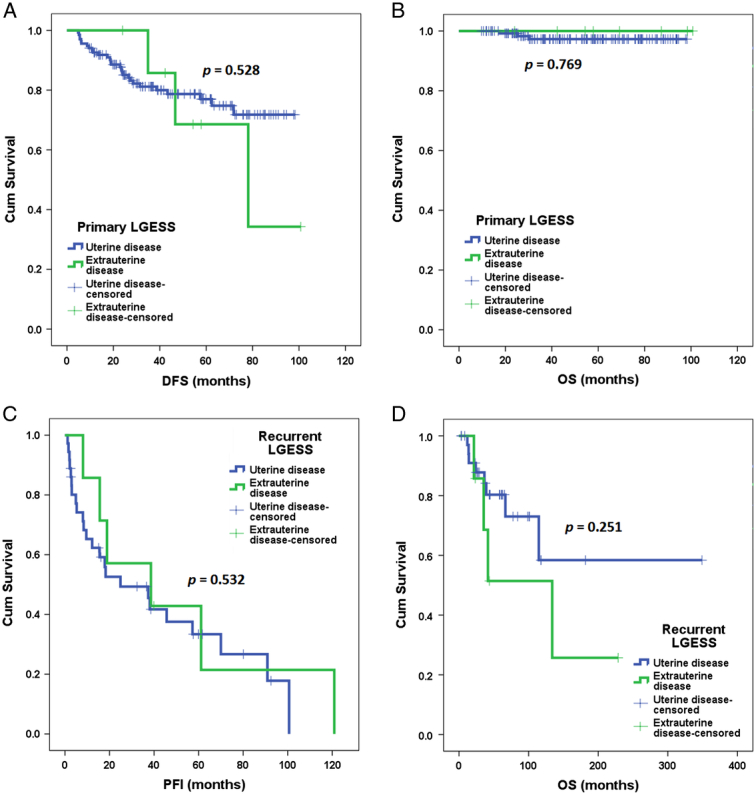
Survival outcomes of patients with low-grade endometrial stromal sarcoma by Kaplan–Meier analysis. (A) Disease-free survival of patients with primary extrauterine or uterine disease. (B) Overall survival (OS) of patients with primary extrauterine or uterine disease. (C) Progression-free interval of patients with recurrent extrauterine or uterine disease. (D) OS of patients with recurrent extrauterine or uterine disease.

**Figure 3:**
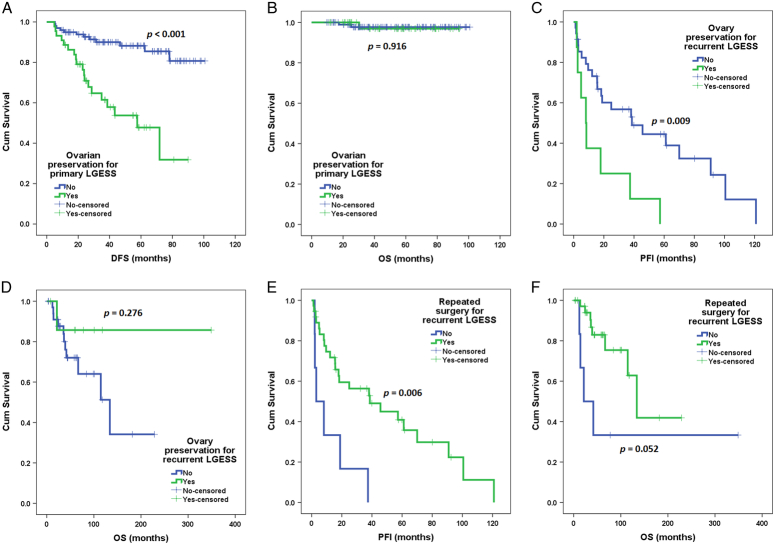
Survival outcomes of patients with low-grade endometrial stromal sarcoma (LGESS) by Kaplan–Meier analysis. (A) Disease-free survival of primary LGESS patients with or without ovarian preservation. (B) Overall survival (OS) of primary LGESS patients with or without ovarian preservation. (C) Progression-free interval (PFI) of recurrent patients with or without ovarian preservation. (D) OS of recurrent patients with or without ovarian preservation. (E) PFI of recurrent patients with or without surgical treatment. (F) OS of recurrent patients with or without surgical treatment.

As shown in Figure 2A and B, in univariate analysis, extrauterine LGESS was not associated with increased risks of recurrence (HR 1.5, 95% CI: 0.4–4.8, *P*=0.528) or mortality (HR 0.04, 0.0-not available, *P*=0.769). As shown in Figure 3A and B, in this population, ovarian preservation was associated with an increased risk of recurrence (HR 4.9, 95% CI: 2.3–10.1, *P*<0.001) but not with mortality (HR 1.1, 95% CI: 0.1–12.6, *P*=0.916). The difference in recurrence remained after adjustment for the presence of extrauterine LGESS. No risk factor was found to be associated with OS. No patient with extrauterine LGESS achieved successful conception in this population. Among the four patients with vaginal LGESS, three underwent vaginal tumor resection, and two had disease relapse later. One patient with extrauterine LGESS (LGE0011) had fertility spared and attempted conception, but unfortunately, she experienced recurrence before successful conception.

### Survival outcomes of LGESS after first recurrence

In patients with recurrent uterine and extrauterine LGESS, 25 of 36 (69.4%) and 5 of 7 (71.4%) developed recurrence, and 8 (22.2%) and 4 (57.1%) died, respectively. The median PFI after the last treatment for recurrence and the OS of patients with recurrent LGESS after the diagnosis of recurrence were 18.3 (range 1–121) and 43.6 (3–349) months, respectively.

### Survival difference between uterine and extrauterine LGESS in recurrent cases

As shown in Figures 2C and D, in univariate analysis, extrauterine LGESS was not associated with increased risks of repeated recurrence (HR 0.7, 95% CI: 0.3–1.9, *P*=0.532) or mortality (HR 2.0, 95% CI: 0.6–7.0, *P*=0.251). As shown in Figures 3C and D, in this population, ovarian preservation was associated with an increased risk of recurrence (HR 3.1, 95% CI: 1.3–7.3, *P*=0.009) but not with mortality (HR 0.3, 95% CI: 0.04–2.5, *P*=0.276). In addition, as shown in Figure 3E and F, repeated surgical treatment for recurrence was associated with decreased risks of recurrence (HR 0.2, 95% CI: 0.1–0.7, *P*=0.006) but not mortality (HR 0.3, 95% CI: 0.1–1.0, *P*=0.052). These differences in recurrence still existed after adjustments for the presence of extrauterine LGESS. No risk factor was found to be associated with OS. No patient with extrauterine LGESS achieved successful conception in this population.

## Discussion

In this cohort study, we compared the clinical characteristics and survival outcomes of patients with uterine and extrauterine LGESS who had primary or recurrent disease. The comparisons revealed that the prognoses of extrauterine LGESS were similar to those of their uterine counterparts. To our knowledge, although this is not the largest cohort of extrauterine LGESS in the literature, it is the first to compare the survival outcomes of extrauterine LGESS to those of uterine LGESS with long-term follow-up. We also provide information for patients with recurrent disease for the first time. Since the tumor behavioral characteristics of extrauterine LGESS are similar to those of uterine LGESS, ovarian preservation is not recommended for older patients not desiring fertility preservation, especially in recurrent situations, as it would significantly increase the risk of recurrence^11,12^. As with uterine LGESS, surgical treatment is of great importance in the management of extrauterine LGESS, for both the primary and recurrent disease states. This study brings to light previously unreported prognostic features that add to the body of knowledge with respect to extrauterine LGESS and may aid in the clinical management of this rare disease.

Primary extrauterine ESS is a rare entity and consists of low-grade and high-grade types^5,6^. In our study, extrauterine disease made up 5.6% of cases in the primary LGESS cohort. Many case reports have discussed LGESS originating from the vagina^8,13,14^, cervix^15,16^, ovaries^5,7,17^, placenta^18^, stomach^19^, ileum^20^, colorectum^21–23^, omentum^24^, sciatic nerves^25^, retroperitoneal cavity^26^, and nonspecific sites within the pelvic cavity^27^. The most common sites involved are the ovaries^5^. Many cases may have nonspecific origin sites within the abdominal and/or pelvic cavity^5,6^. These findings suggest that the identification and differentiation of extrauterine LGESS are of clinical importance, as LGESS often has an indolent course with a better prognosis than other ovarian sarcomas, indicating the importance of a correct diagnosis^7^.

The differential diagnosis of extrauterine LGESS involves primarily metastatic uterine sarcoma of the same cell type. One fundamental element of good pathology practice, namely, awareness of the clinical history and knowledge of the uterine status, is crucial in this regard^7^ and paramount in this distinction. The characteristic gross finding of nodular infiltrative growth with vascular involvement as seen in uterine LGESS is rarely seen within the ovarian parenchyma^6,7^. A number of extrauterine LGESSs have the same genetic alterations as their uterine counterparts, suggesting that extrauterine LGESS has a histogenesis that is similar to that of uterine tumors^28^. Comprehensive pathological review could only be available when hysterectomy is performed, which is critical for young patients desiring fertility. When hysterectomy precedes removal of the extrauterine disease or if the uterus is left in situ, a definitive diagnosis may rely on review of prior pathology material, on review of imaging evaluations, on endometrial sampling if applicable, or on thorough sampling of the extrauterine tumor to assess histological features that could aid in the exclusion of a uterine primary^5^. However, these methods have a high risk of missed diagnosis of primary uterine disease. To rule out the possibility of metastasis from the uterus, we made efforts to review uterine histology, and all extrauterine LGESS patients had direct or indirect evidence supporting an uninvolved uterus. In our study, ovarian preservation led to an increased risk of recurrence, and no extrauterine LGESS patient with an intact uterus achieved successful conception. However, for primary uterine LGESS, fertility-sparing treatment has been reported in many case reports^29–37^. Therefore, uterine-sparing treatment seemed inappropriate for patients suspected of extrauterine LGESS.

An unexpected location and unusual presentation of extrauterine LGESS may make the diagnosis challenging. The differential diagnosis of extrauterine LGESS with other neoplasms in the same sites is fraught with difficulties, particularly given the potential for less common histological patterns that mimic other entities. Additionally, one-quarter of cases could be misdiagnosed at initial treatment^5^. When a primary ovarian LGESS is determined, the differential diagnosis is most often with a sex cord-stromal tumor^5,7^. The immunohistochemical^5^ and genetic characteristics^11,12,38,39^ of the tumor are very important for the differential diagnosis of this rare neoplasm.

Conditions, such as adenosarcoma and endometriosis, should be considered in the differential diagnosis when true glandular/epithelial differentiation is present. In our study, endometriosis and adenomyosis were noted in six (40.0%) and two (13.3%) patients with extrauterine LGESS, respectively. Many reports have suggested endometriosis as the origin of LGESS^9,16,20,22,25,40–43^. Endometriosis was noted in almost half of extrauterine LGESS cases^5^, and endometriotic foci were identified adjacent to the tumor in almost half of ovarian LGESS cases^7^. A history of endometriosis might provide a clue for the differential diagnosis of extrauterine LGESS from other types of sarcomas, which lack such an association^44,45^. When there is an apparent lack of associated endometriosis, it is possible that the extrauterine LGESS originated from gland-poor foci of endometriosis (stromal endometriosis) or that overgrowth of the tumor has obscured the underlying endometriosis^5^.

The observational design and limited sample sizes of our study are its main limitations. Additionally, seven of 15 patients lacked direct evidence of a noninvolved uterus, which would weaken the study power. Due to the limited sample size, we could not sufficiently evaluate other important clinicopathological characteristics, such as tumor size, mitotic index, location, vascular invasion, and stages, in the context of survival outcomes. In a well-designed multicenter study, a stringent sample size estimation should be performed as to guarantee enough statistic power. We did not perform genetic testing although two thirds of LGESS harbor genetic fusions involving polycomb family genes^1^. We did not perform FIGO staging in the extrauterine LGESS, as Masand *et al*.^5^ observed, the conditions of patients with multifocal disease were not worse than those of patients with single-site disease, suggesting that multifocal disease does not represent advanced-stage disease in extrauterine LGESS. The optimal adjuvant therapy after surgery remains unclear. Besides, histological characteristics could provide prognostic information for LGESS^46^.

## Conclusions

Extrauterine LGESS had similar clinical characteristics and survival outcomes to those of uterine LGESS in both the primary or recurrent disease states, and surgical treatment is the choice for all types of LGESS to achieve improved survival. Ovarian preservation would be detrimental to DFS but not to OS.

## Ethics approval and registration

The Institutional Review Board of Peking Union Medical College Hospital has approved this study (No. SK-1289). The trial was registered on ClinicalTrials.gov with the identifier NCT05310318.

## Consent for publication

Consents for publication have been obtained from all patients.

## Sources of funding

This study is supported by the State Key Laboratory for Complex, Severe and Rare Diseases in Peking Union Medical College Hospital, by the Beijing Science and Technology Projects (No. Z211100002921068), by the Key Research Project of Beijing Natural Science Foundation (No. Z220013), by the CAMS Innovation Fund for Medical Sciences (CIFMS) (No. 2022-I2M-C&T-B-033), by the National High Level Hospital Clinical Research Funding (No. 2022-PUMCH-A-117, 2022-PUMCH-B-083, 2022-PUMCH-C-010, 2022-PUMCH-C-022 and 2022-PUMCH-D-003), by the Le Fund (No. KH-2020-LJJ-004, 034 and 035), by the Beijing CSCO Research Fund for Clinical Oncology (No. Y-QL2019-0165 and Y-zai2021/ms-0198), and by the China Postdoctoral Science Foundation (No. 2022T150066). The funders had no role in the study design, data collection and analysis, decision to publish, or preparation of the manuscript.

## Author contribution

L.L.: conceived of the original idea for the study, interpreted results, carried out the statistical analysis, edited the paper and was overall guarantor; Q.D.: obtained ethical approval, contributed to the preparation of the data set, interpreted results, and contributed to drafts of the paper; B.X.: contributed to the study design, interpretation of results, and commented on drafts of the paper; Y.Y. and H.W.: conducted the pathological evaluation. All authors have approved the final version of the manuscript.

## Conflicts of interest disclosure

All authors declare that they have no conflicts of interest to disclose.

## Research registration unique identifying number (UIN)

ClinicalTrials.gov ID: NCT05310318.

## Guarantor

The author Lei Li is the guarantor.

## Availability of data and material

All data of this study has been contained in the supplement file.

Data Availability: The data supporting the findings of this study are available from the corresponding author upon request.

Data Statement: The data that support the findings of this study are available from the corresponding author upon reasonable request. The data are not publicly available due to privacy or ethical restrictions.

## Provenance and peer review

This paper was not invited.

## Statement of submission

The paper is not under consideration by another journal, and the results presented in this work have not been previously presented or published.

## Data source

The data used in this study were obtained from the clinicopathological data.

## Data format

The data are available in Excel.

## Data access duration

The data will be available for 5 years after the publication of this article.

## Data custodian contact

For data requests, please contact the corresponding author, Li Lei, via e-mail: lileigh@163.com.

## Supplementary Material

**Figure:**
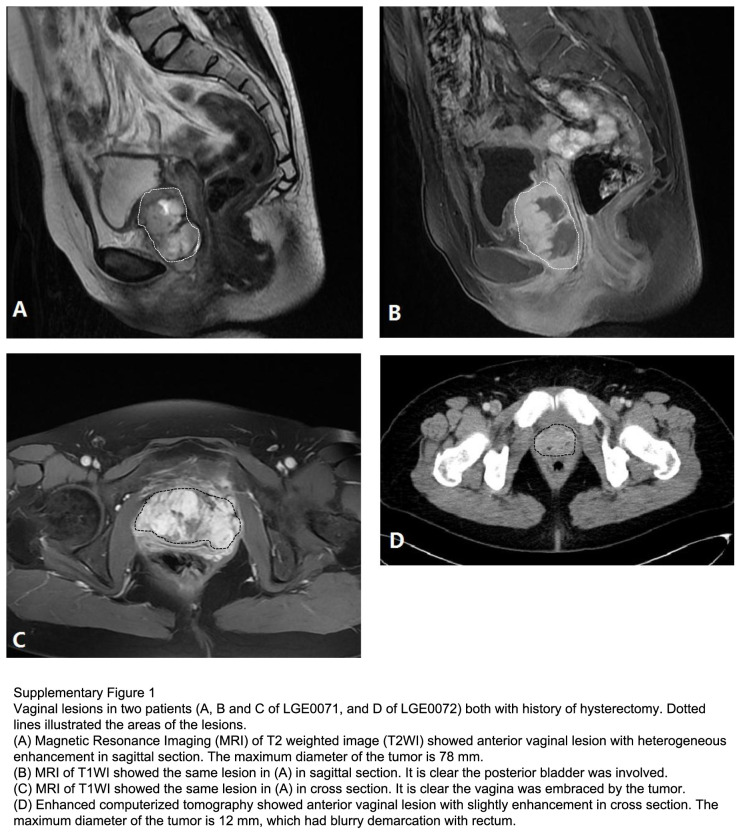


**Figure:**