# Supplementary material for: Prognosis of uterine and extrauterine low-grade endometrial stromal sarcoma: an observational cohort study
Source: Int J Surg. 2024 Feb 7;110(4):1919–28. doi: 10.1097/JS9.0000000000001146 (PMC11020013; doi:10.1097/JS9.0000000000001146)
Supplement: Supplementary file 1 [file js9-110-1919-s001.pdf]

No	Extrauterine LGESS	LVSI	ER(+)	PR(+)	MI>5	Definite mitotic	Ovarian preservation	Fertility sparing	Recurrence	Mortality	DFS (months)
LGE0001	Vaginal	No	Yes	Yes	No	3-5	No	No	Yes	No	47
LGE0002	Vaginal	No	Yes	Yes	No	1-3	No	No	No	No	24
LGE0004	Vaginal	No	Yes	Yes	No	1-3	No	No	No	No	54
LGE0006	Vaginal	No	Yes	Yes	No	1-3	Yes	Yes	Yes	No	35
LGE0007	Unknown	No	Yes	Yes	Yes	>10	No	No	Yes	No	78
LGE0009	Unknown		Yes	No	No	3-5	No	No	No	No	101
LGE0011	Residual cervical	No	Yes	Yes	No	1-3	Yes	No	No	No	42
LGE0012	Ovarian	No	Yes	Yes	No	1-3	No	No	No	No	58
LGE0017		No	Yes	Yes	Yes	6-10	No	No	Yes	Yes	6
LGE0019		No	Yes	Yes	No	1-3	No	No	Yes	No	27
LGE0020		No	Yes	Yes	No	3-5	No	No	Yes	No	6
LGE0021		No	Yes	Yes	Yes	5-10	No	No	No	No	87
LGE0022		No	Yes	No	No	3-5	No	No	Yes	No	31
LGE0023		No	Yes	Yes	No	3-5	No	No	Yes	Yes	11
LGE0026		No	Yes	Yes	No	3-5	No	No	No	No	30
LGE0030			Yes	Yes	Yes	5-10	No	No	No	No	98
LGE0032		No	Yes	Yes	No	3-5	No	No	No	No	34
LGE0035		No	Yes	Yes	No	1-3	No	No	No	No	58
LGE0039		No	No	Yes	No	3-5	No	No	No	No	14
LGE0040		No	Yes	Yes	No	3-5	No	No	No	No	76
LGE0041		No	Yes	Yes	No	3-5	No	No	No	No	91
LGE0044		No	Yes	Yes	No	1-3	No	No	No	No	62
LGE0045		No	Yes	Yes	No	3-5	No	No	No	No	20
LGE0047		Yes	Yes	Yes	No	1-3	No	No	Yes	No	19
LGE0051		Yes	Yes	Yes	Yes	>10	No	No	Yes	No	62
LGE0053		Yes	Yes	Yes	No	3-5	No	No	No	No	71
LGE0054		No	Yes	No	No	<1	No	No	Yes	No	9
LGE0055		No	Yes	Yes	Yes	>10	No	No	Yes	No	23
LGE0057		No	Yes	Yes	No	<1	No	No	Yes	No	5
LGE0058		Yes	Yes	Yes	No	1-3	No	No	No	No	77
LGE0059		No	Yes	Yes	No	1-3	No	No	No	No	76
LGE0061		Yes	Yes	Yes	Yes	>10	No	No	No	No	30
LGE0062		No	Yes	No	No	1-3	No	No	No	No	44
LGE0063		Yes	Yes	Yes	No	3-5	No	No	No	No	17
LGE0064		Yes	Yes	Yes	No	<1	No	No	No	No	27
LGE0065		Yes	Yes	Yes	No	1-3	No	No	No	No	36
LGE0066		No	Yes	Yes	No	3-5	No	No	No	No	71
LGE0067		No	Yes	Yes	No	3-5	No	No	No	No	83
LGE0068		No	Yes	No	No	3-5	No	No	No	No	55
LGE0069			Yes	Yes	No	3-5	No	No	No	No	48
LGE0070			Yes	Yes	Yes	5-10	No	No	No	No	95
LGE0071		No	Yes	No	No	1-3	No	No	No	No	70
LGE0072		Yes	Yes	Yes	Yes	5-10	No	No	No	No	23

LGE0073	No	Yes	Yes	No	3-5	No	No	No	No	15
LGE0074	No	Yes	Yes	No	1-3	No	No	No	No	71
LGE0075	Yes	Yes	Yes	No	<1	No	No	No	No	35
LGE0076	No	Yes	Yes	Yes	5-10	No	No	No	No	39
LGE0077	Yes	No	Yes	No	3-5	No	No	No	No	25
LGE0078	No	No	Yes	No	1-3	No	No	No	No	33
LGE0079	No	Yes	Yes	No	3-5	No	No	No	No	14
LGE0080		Yes	Yes	No	3-5	No	No	No	No	48
LGE0081		Yes	Yes	Yes	5-10	No	No	No	No	42
LGE0082		Yes	Yes	No	3-5	No	No	No	No	48
LGE0083	Yes	Yes	Yes	No	3-5	No	No	No	No	67
LGE0084	No	Yes	No	No	1-3	No	No	No	No	25
LGE0085	No	Yes	Yes	No	3-5	No	No	No	No	98
LGE0086		Yes	Yes	No	1-3	No	No	No	No	59
LGE0087	No	Yes	Yes	Yes	5-10	No	No	No	No	28
LGE0088	No	Yes	Yes	No	1-3	No	No	No	No	93
LGE0089	No	Yes	Yes	Yes	5-10	No	No	No	No	62
LGE0090	Yes	Yes	Yes	No	1-3	No	No	No	No	13
LGE0091	Yes	Yes	Yes	Yes	5-10	No	No	No	No	32
LGE0092	No	Yes	Yes	Yes	>10	No	No	No	No	31
LGE0093	Yes	Yes	Yes	No	3-5	No	No	No	No	37
LGE0094	Yes	No	Yes	No	1-3	No	No	No	No	10
LGE0095	No	Yes	Yes	No	3-5	No	No	No	No	62
LGE0096	No	Yes	Yes	No	1-3	No	No	No	No	69
LGE0097	No	Yes	Yes	No	1-3	No	No	No	No	72
LGE0098		Yes	Yes	No	1-3	No	No	No	No	79
LGE0099	Yes	Yes	No	No	3-5	No	No	No	No	60
LGE0101	No	Yes	No	No	3-5	No	No	No	No	20
LGE0102	No	Yes	Yes	Yes	5-10	No	No	No	No	45
LGE0103	No	Yes	Yes	No	1-3	No	No	No	No	37
LGE0104	No	No	Yes	No	1-3	No	No	No	No	89
LGE0105	Yes	No	Yes	No	1-3	No	No	No	No	36
LGE0106	Yes	Yes	Yes	No	3-5	No	No	No	No	14
LGE0107	No	Yes	Yes	No	1-3	No	No	No	No	88
LGE0108	No	Yes	Yes	No	1-3	No	No	No	No	30
LGE0109	No	No	Yes	Yes	>10	No	No	No	No	67
LGE0110		Yes	No	Yes	5-10	No	No	No	No	51
LGE0111		Yes	No	Yes	5-10	No	No	No	No	58
LGE0112	No	Yes	Yes	No	3-5	No	No	No	No	10
LGE0113	No	Yes	No	No	3-5	No	No	No	No	85
LGE0114	No	Yes	Yes	No	3-5	No	No	No	No	85
LGE0115		Yes	Yes	No	1-3	No	No	No	No	33
LGE0116		Yes	Yes	No	1-3	No	No	No	No	39
LGE0117	No	Yes	Yes	Yes	5-10	No	No	No	No	78
LGE0118	No	Yes	Yes	No	3-5	No	No	No	No	12

LGE0119	No	Yes	No	No	3-5	No	No	No	No	27
LGE0120	Yes	Yes	No	No	1-3	No	No	No	No	23
LGE0121	No	Yes	Yes	No	1-3	No	No	No	No	24
LGE0122	No	Yes	Yes	Yes	5-10	No	No	No	No	73
LGE0123	No	Yes	Yes	Yes	5-10	No	No	No	No	41
LGE0124	No	Yes	Yes	No	1-3	No	No	No	No	62
LGE0125	No	Yes	Yes	No	<1	No	No	No	No	48
LGE0126	No	No	Yes	No	3-5	No	No	No	No	85
LGE0127	No	Yes	Yes	No	3-5	No	No	No	No	53
LGE0128		Yes	Yes	No	1-3	No	No	No	No	78
LGE0129	No	Yes	Yes	Yes	>10	No	No	No	No	82
LGE0130	No	Yes	No	No	3-5	No	No	No	No	85
LGE0131	No	Yes	Yes	No	3-5	No	No	No	No	45
LGE0132	No	Yes	Yes	No	3-5	Yes	No	Yes	No	43
LGE0133	Yes	Yes	No	No	3-5	Yes	No	Yes	No	14
LGE0134	No	Yes	Yes	No	1-3	Yes	No	Yes	No	24
LGE0135	No	Yes	Yes	No	3-5	Yes	No	Yes	No	29
LGE0136	Yes	Yes	Yes	No	1-3	Yes	No	Yes	No	24
LGE0137	No	No	Yes	No	3-5	Yes	No	Yes	No	11
LGE0138	Yes	Yes	Yes	No	3-5	Yes	No	Yes	No	5
LGE0139	No	Yes	Yes	Yes	6-10	Yes	No	Yes	Yes	6
LGE0140	No	Yes	Yes	No	1-3	Yes	No	No	No	23
LGE0141		Yes	Yes	No	3-5	Yes	No	No	No	63
LGE0142	No	No	Yes	No	3-5	Yes	No	No	No	81
LGE0143	Yes	Yes	Yes	No	1-3	Yes	No	No	No	62
LGE0144	No	No	Yes	Yes	>10	Yes	No	No	No	37
LGE0145	No	Yes	Yes	No	1-3	Yes	No	No	No	12
LGE0146	No	Yes	No	No	1-3	Yes	No	No	No	20
LGE0147	No	Yes	Yes	No	3-5	Yes	No	No	No	43
LGE0148	No	Yes	Yes	No	1-3	Yes	No	No	No	12
LGE0149	No	Yes	Yes	No	1-3	Yes	No	No	No	21
LGE0150		Yes	Yes	No	3-5	Yes	No	No	No	58
LGE0151	No	Yes	Yes	No	3-5	Yes	No	No	No	40
LGE0152	No	Yes	Yes	No	3-5	Yes	No	No	No	59
LGE0153	No	Yes	Yes	No	3-5	Yes	Yes	Yes	No	39
LGE0154	No	Yes	No	No	<1	Yes	Yes	Yes	No	72
LGE0155	No	Yes	Yes	No	3-5	Yes	Yes	Yes	No	19
LGE0156	No	Yes	Yes	No	1-3	Yes	Yes	Yes	No	23
LGE0157	No	Yes	Yes	Yes	6-10	Yes	Yes	Yes	No	9
LGE0158	No	Yes	Yes	Yes	6-10	Yes	Yes	Yes	No	19
LGE0159	No	Yes	Yes	No	1-3	Yes	Yes	Yes	No	17
LGE0160	Yes	No	Yes	Yes	>10	Yes	Yes	Yes	No	6
LGE0161	No	Yes	Yes	No	1-3	Yes	Yes	Yes	No	26
LGE0162	No	Yes	No	No	3-5	Yes	Yes	Yes	No	58
LGE0163	No	Yes	Yes	Yes	5-10	Yes	Yes	No	No	57

LGE0164	No	Yes	No	No	1-3	Yes	Yes	No	No	25
LGE0165	No	Yes	Yes	No	3-5	Yes	Yes	No	No	55
LGE0166	No	Yes	Yes	No	3-5	Yes	Yes	No	No	21
LGE0167	No	Yes	Yes	No	1-3	Yes	Yes	No	No	66
LGE0168	No	Yes	Yes	No	3-5	Yes	Yes	No	No	55
LGE0169	No	Yes	Yes	Yes	5-10	Yes	Yes	No	No	90
LGE0170	No	Yes	Yes	Yes	5-10	Yes	Yes	No	No	25
LGE0171	No	Yes	Yes	No	1-3	Yes	Yes	No	No	37
LGE0172	No	Yes	Yes	No	1-3	Yes	Yes	No	No	29
LGE0173	No	Yes	Yes	No	1-3	Yes	Yes	No	No	25
LGE0012										
LGE0071										
LGE0072										
LGE0011										
LGE0017										
LGE0070										
LGE0068										
LGE0069										
LGE0051										
LGE0053										
LGE0055										
LGE0057										
LGE0058										
LGE0061										
LGE0102										
LGE0124										
LGE0130										
LGE0131										
LGE0132										
LGE0137										
LGE0161										
LGE0162										
LGE0173										
LGE0044										
LGE0045										
LGE0047										
LGE0054										
LGE0059										
LGE0062										
LGE0092										
LGE0093										
LGE0094										
LGE0095										
LGE0096										
LGE0097										

LGE0098
LGE0099
LGE0101
LGE0103
LGE0104
LGE0105
LGE0106
LGE0107
LGE0108
LGE0109
LGE0110
LGE0111
LGE0112
LGE0113
LGE0114
LGE0115
LGE0116
LGE0117
LGE0118
LGE0119
LGE0120
LGE0121
LGE0122
LGE0123
LGE0125
LGE0126
LGE0127
LGE0128
LGE0129
LGE0133
LGE0134
LGE0135
LGE0136
LGE0138
LGE0139
LGE0140
LGE0141
LGE0142
LGE0143
LGE0144
LGE0145
LGE0146
LGE0147
LGE0148
LGE0149

LGE0150
LGE0151
LGE0152
LGE0153
LGE0154
LGE0155
LGE0156
LGE0157
LGE0158
LGE0159
LGE0160
LGE0163
LGE0164
LGE0165
LGE0166
LGE0167
LGE0168
LGE0169
LGE0170
LGE0171
LGE0172
LGE0026
LGE0039
LGE0009
LGE0030
LGE0032
LGE0035
LGE0040
LGE0041
LGE0079
LGE0080
LGE0081
LGE0082
LGE0083
LGE0084
LGE0085
LGE0086
LGE0087
LGE0088
LGE0089
LGE0090
LGE0091
LGE0001
LGE0002
LGE0004

LGE0006
LGE0007
LGE0019
LGE0020
LGE0021
LGE0022
LGE0023
LGE0063
LGE0064
LGE0065
LGE0066
LGE0067
LGE0073
LGE0074
LGE0075
LGE0076
LGE0077
LGE0078

	**OS (months)**	**Surgical route**	**Uterine pathology**	**Age at diagnosis**	**Age groups**	**Menopause**	**Gravidity**	**Parity**	**Attempting pregnancy**	**Interval to concepti**	**Conceptive methods**
LGE0001	88	Open		26	1	No	1	1	Yes	12	Natural
LGE0002	94	Hys		28	1	No	1	1	Yes	14	Natural
LGE0004	69	Open		25	1	No	3	2	Yes	10	Natural
LGE0006	43	Open		29	1	No	1	0	Yes		
LGE0007	66	Open		37	2	No	0	0	No		
LGE0009	94	Open		33	2	No	4	1			
LGE0011	69	Vagina	Normal	36	2	No	3	2			
LGE0012	87	Vagina	Normal	48	2	No	3	1			
LGE0017	98	Open	Myoma	50	2	Yes	3	1			
LGE0019	43	Lap		35	2	No	0	0	No		
LGE0020	53	Open		39	2	No	2	1	No		
LGE0021	58	Open		16	1	No	0	0	No		
LGE0022	77	Hys		35	2	No	1	1	No		
LGE0023	58	Hys		20	1	No	0	0	No		
LGE0026	79	Open		52	3	Yes	7	2			
LGE0030	78	Open		35	2	No	1	1			
LGE0032	24	Open		28	1	No	1	1			
LGE0035	79	Open		44	2	No	5	4			
LGE0039	54	Lap		50	2	No	3	1			
LGE0040	24	Lap		46	2	No	4	2			
LGE0041	30	Lap		36	2	No	2	1			
LGE0044	42	Lap		35	2	No	3	0			
LGE0045	92	Lap		45	2	No	1	1			
LGE0047	71	Open		41	2	No	4	1			

LGE0051	17	Open		52	3	Yes	0	0			
LGE0053	28	Lap		65	3	Yes	1	1			
LGE0054	30	Lap		45	2	No	2	1			
LGE0055	60	Open		50	2	Yes	1	1			
LGE0057	87	Lap		55	3	Yes	1	1			
LGE0058	61	Open		55	3	Yes	3	3			
LGE0059	54	Open		49	2	No	1	1			
LGE0061	25	Open		58	3	Yes	5	5			
LGE0062	47	Lap		48	2	No	3	1			
LGE0063	57	Hys		33	2	No	1	0	Yes	11	Natural
LGE0064	25	Lap		33	2	No	0	0	Yes	10	IVF
LGE0065	55	Hys		26	1	No	0	0	Yes	20	Natural
LGE0066	21	Hys		20	1	No	0	0	Yes	10	Natural
LGE0067	66	Hys		37	2	No	2	1	Yes		
LGE0068	42	Vagina	Myoma	53	3	Yes	3	3			
LGE0069	58	Open	Adenomyosis	30	1	No	0	0			
LGE0070	101	Open	Myoma	61	3	Yes	5	2			
LGE0071	24	Vagina	Adenomyosis	36	2	No	2	2			
LGE0072	54	Open	Myoma	45	2	No	2	1			
LGE0073	55	Lap		33	2	No	5	1	No		
LGE0074	90	Hys		15	1	No	0	0	No		
LGE0075	25	Hys		37	2	No	3	2	No		
LGE0076	37	Hys		25	1	No	0	0	No		
LGE0077	29	Lap		23	1	No	0	0	No		
LGE0078	25	Lap		40	2	No	1	1	No		
LGE0079	23	Lap		33	2	No	1	0			
LGE0080	63	Open		37	2	No	0	0			
LGE0081	81	Lap		29	1	No	0	0			
LGE0082	62	Lap		29	1	No	0	0			
LGE0083	37	Lap		35	2	No	1	1			
LGE0084	12	Lap		41	2	No	0	0			
LGE0085	20	Lap		27	1	No	2	2			
LGE0086	43	Lap		36	2	No	0	0			
LGE0087	12	Lap		25	1	No	0	0			
LGE0088	21	Lap		34	2	No	5	2			
LGE0089	58	Lap		41	2	No	1	1			
LGE0090	40	Lap		43	2	No	1	0			
LGE0091	59	Lap		31	2	No	1	0			
LGE0092	77	Open		35	2	No	0	0			
LGE0093	76	Open		49	2	No	4	1			
LGE0094	30	Open		49	2	No	2	2			
LGE0095	44	Lap		41	2	No	4	1			
LGE0096	17	Lap		49	2	No	0	0			
LGE0097	27	Open		51	3	No	0	0			
LGE0098	36	Open		43	2	No	1	1			

LGE0099	71	Lap	45	2	No	2	2
LGE0101	83	Open	49	2	No	2	1
LGE0102	30	Open	64	3	Yes	0	0
LGE0103	55	Open	44	2	No	0	0
LGE0104	48	Lap	34	2	No	3	1
LGE0105	95	Open	47	2	No	4	1
LGE0106	70	Open	39	2	No	3	1
LGE0107	23	Open	50	2	No	4	2
LGE0108	15	Open	42	2	No	3	2
LGE0109	71	Open	38	2	No	3	1
LGE0110	35	Open	33	2	No	4	2
LGE0111	39	Open	21	1	No	1	1
LGE0112	25	Open	33	2	No	1	1
LGE0113	33	Lap	50	2	No	3	1
LGE0114	14	Open	43	2	No	3	1
LGE0115	48	Open	43	2	No	4	0
LGE0116	42	Open	35	2	No	1	1
LGE0117	48	Open	50	2	No	2	2
LGE0118	67	Open	34	2	No	3	2
LGE0119	25	Open	46	2	No	4	2
LGE0120	98	Lap	49	2	No	6	3
LGE0121	59	Lap	44	2	No	3	1
LGE0122	28	Lap	43	2	No	3	1
LGE0123	93	Open	22	1	No	0	0
LGE0124	98	Lap	52	3	Yes	4	1
LGE0125	62	Open	38	2	No	1	1
LGE0126	13	Lap	48	2	No	3	1
LGE0127	32	Lap	44	2	No	3	4
LGE0128	31	Lap	36	2	No	2	1
LGE0129	37	Open	44	2	No	0	0
LGE0130	34	Lap	65	3	Yes	1	1
LGE0131	58	Lap	51	3	Yes	4	2
LGE0132	14	Lap	53	3	Yes	1	1
LGE0133	10	Open	47	2	No	1	1
LGE0134	62	Lap	44	2	No	6	1
LGE0135	69	Lap	45	2	No	1	1
LGE0136	72	Open	47	2	No	4	1
LGE0137	76	Open	51	3	Yes	4	1
LGE0138	79	Lap	49	2	No	2	1
LGE0139	60	Open	29	1	No	2	0
LGE0140	20	Lap	46	2	No	3	2
LGE0141	45	Lap	47	2	No	2	2
LGE0142	37	Lap	42	2	No	6	2
LGE0143	89	Open	35	2	No	2	1
LGE0144	36	Open	29	1	No	1	1

LGE0145	14	Open	43	2	No	0	0
LGE0146	88	Open	42	2	No	1	1
LGE0147	30	Open	49	2	No	2	1
LGE0148	67	Open	37	2	No	5	1
LGE0149	51	Open	33	2	No	2	1
LGE0150	58	Lap	41	2	No	5	2
LGE0151	10	Open	46	2	No	2	1
LGE0152	85	Open	48	2	No	4	1
LGE0153	85	Lap	44	2	No	1	1
LGE0154	33	Lap	47	2	No	3	1
LGE0155	39	Lap	31	2	No	2	1
LGE0156	78	Lap	51	3	No	2	1
LGE0157	12	Lap	37	2	No	2	1
LGE0158	27	Lap	45	2	No	3	1
LGE0159	23	Lap	41	2	No	1	1
LGE0160	24	Open	46	2	No	1	1
LGE0161	91	Lap	51	3	Yes	3	1
LGE0162	62	Open	53	3	Yes	4	3
LGE0163	73	Open	48	2	No	1	1
LGE0164	41	Lap	46	2	No	3	1
LGE0165	62	Lap	44	2	No	12	2
LGE0166	48	Lap	35	2	No	1	1
LGE0167	85	Lap	47	2	No	3	1
LGE0168	53	Open	45	2	No	3	1
LGE0169	78	Lap	43	2	No	6	2
LGE0170	82	Open	49	2	No	2	1
LGE0171	85	Open	41	2	No	6	1
LGE0172	45	Lap	48	2	No	3	1
LGE0173	20	Lap	68	3	Yes	0	0

	**Reasons of not attempting pregnancy**	**Pregnant time**	**Abortion times**	**Number of live birth**	**Live birth weeks**	**Complications during pregnancy**	**Treatment after recurrence**	**Personal cancer history**	**Family cancer history**	**Max diameter of tumor**	**FIGO stage**
LGE0001		1	0	1	38		Accepted non-fertility sparing surgeries after		Uncle, stomach	50	I
LGE0002		1	0	1	38		Accepted non-fertility sparing surgeries after				IA
LGE0004		2	0	2	40		Accepted repeated fertility sparing				I
LGE0006							Accepted non-fertility sparing surgeries			100	IIIB
LGE0007	During treatment						Accepted non-fertility sparing surgeries after			140	IIB
LGE0009										80	IIB
LGE0011										30	
LGE0012									Sister, lung	40	
LGE0017										147	
LGE0019	Unmarried						Accepted non-fertility sparing surgeries			46	I
LGE0020	None						Accepted non-fertility sparing surgeries			80	IIIB
LGE0021	Unmarried						Accepted repeated fertility sparing			43	IC
LGE0022	None						Accepted non-fertility sparing surgeries				I
LGE0023	Unmarried						Accepted repeated fertility sparing			70	I

LGE0026								60	IC
LGE0030								80	I
LGE0032								73	IIB
LGE0035									I
LGE0039									IC
LGE0040									IIA
LGE0041							Aunt, breast:	43	IB
LGE0044								13	IC
LGE0045								59	IC
LGE0047								99	IIA
LGE0051									IIA
LGE0053								50	I
LGE0054							Father, duodenum	86	I
LGE0055								57	I
LGE0057									I
LGE0058									IC
LGE0059								170	IC
LGE0061							Mother, liver; sisiter	61	I
LGE0062								40	IB
LGE0063		1	0	1	38	Hypertension		30	I
LGE0064		1	0	1	36	Preterm		60	I
LGE0065		1	0	1	38				I
LGE0066		1	0	1	38			37	I
LGE0067								30	I
LGE0068								29	
LGE0069								103	
LGE0070								102	
LGE0071								78	
LGE0072								35	
LGE0073	During treatment							50	I
LGE0074	Unmarried								I
LGE0075	None							60	I
LGE0076	Unmarried							60	I
LGE0077	Unmarried							145	I
LGE0078	None							55	I
LGE0079								42	I
LGE0080								130	I
LGE0081								60	I
LGE0082								97	I
LGE0083								60	IIB
LGE0084								50	I
LGE0085							Mother, unterine	18	I
LGE0086									I
LGE0087									IB
LGE0088									I

LGE0089		Father, pharynx	80	I
LGE0090			68	I
LGE0091			30	I
LGE0092		Grandmoth er, uterine		IIB
LGE0093			40	IIB
LGE0094			80	IIB
LGE0095				I
LGE0096				IC
LGE0097				IC
LGE0098			105	IVA
LGE0099				IIA
LGE0101			80	IIB
LGE0102			70	IIA
LGE0103			66	IIA
LGE0104			54	I
LGE0105			35	I
LGE0106				I
LGE0107			91	IVB
LGE0108			88	I
LGE0109			80	IV
LGE0110			64	I
LGE0111				I
LGE0112		Grandmoth er, lung;	35	IIA
LGE0113			48	IIB
LGE0114	Thyroid		123	IIIB
LGE0115			117	IIB
LGE0116			100	IVB
LGE0117			97	IVB
LGE0118			96	IIB
LGE0119			75	I
LGE0120		Mother, stomach	74	I
LGE0121			63	I
LGE0122			63	I
LGE0123			60	I
LGE0124			60	I
LGE0125			53	IC
LGE0126			45	I
LGE0127			30	I
LGE0128			68	I
LGE0129			111	IIB
LGE0130			72	IC
LGE0131	Breast	Sister, uterine	60	I
LGE0132			60	I
LGE0133			80	IC
LGE0134				IC

LGE0135		Father, lung		I
LGE0136		Father, stomach		I
LGE0137				I
LGE0138				I
LGE0139				I
LGE0140				I
LGE0141		Father, liver		IB
LGE0142		Grandfather, stomach;		IB
LGE0143				IIA
LGE0144				IVB
LGE0145				IVB
LGE0146			140	IIB
LGE0147			120	I
LGE0148			80	IIIB
LGE0149			80	I
LGE0150			80	I
LGE0151			78	IC
LGE0152			74	I
LGE0153			69	I
LGE0154			67	I
LGE0155			66	I
LGE0156			61	I
LGE0157		Father, liver	60	I
LGE0158	Breast		60	I
LGE0159			60	I
LGE0160			50	I
LGE0161			50	I
LGE0162			50	I
LGE0163			50	I
LGE0164			50	I
LGE0165			43	II
LGE0166			42	I
LGE0167			40	I
LGE0168		Father, colon	40	IB
LGE0169			34	I
LGE0170			32	I
LGE0171			30	I
LGE0172			26	I
LGE0173			20	I

	**Retroperitoneal lymphadenectomy**	**Metastasis to retroperit**	**Hormone treatment**	**Chemotherapy**	**Radiotherapy**	**Hormone regimens**	**Hormone treatment periods (months)**	**Chemotherapy regimens**	**Chemotherapy courses**	**Recurrent sites**	**Birth date**
LGE0001	No		No	Yes	No			PEI	4	Uterus, lung	1986/10/1

LGE0002	No		Yes	No	No	Progestin	12				Uterus, [illegible]	1984/7/21
LGE0004	No		No	No	No						Retrospective	1989/10/21
LGE0006	No		No	No	No						Pelvic and	1988/4/9
LGE0007	No		No	No	No						Pelvic cavity	1978/1/25
LGE0009	No		No	No	No						Pelvic cavity	1979/4/23
LGE0011	No		No	No	No						Vagina	1978/10/27
LGE0012	No		No	No	No						Vagina	1964/11/29
LGE0017	No		Yes	No	No	Aromatase inhibitor	30				Pelvic and	1962/11/1
LGE0019	No		Yes	No	No	GnRHa	6				Uterus	1981/12/8
LGE0020	No		Yes	No	No	Progestin	9				Pelvic and	1976/10/25
LGE0021	Yes	No	Yes	No	No	Progestin	6				Pelvic cavity	1998/9/29
LGE0022	No		No	No	No						Uterus	1978/10/7
LGE0023	No		No	No	No						Uterus	1995/5/2
LGE0026	No		No	Yes	No				Docetaxel/gemcitabine	4	Pelvic cavity,	1961/4/22
LGE0030	Yes	Yes	Yes	No	No	Progestin	6				Ovaries, parametri	1978/7/22
LGE0032	No		Yes	No	No	Progestin	12				Vaginal stump	1990/2/16
LGE0035	No		No	No	No						Vaginal stump	1969/5/20
LGE0039	No		No	No	No						Vaginal stump	1966/2/27
LGE0040	No		No	No	No						Vaginal stump	1971/9/30
LGE0041	Yes	No	No	No	No						Pelvic cavity	1979/3/12
LGE0044	No		Yes	No	Yes	Progestin	19				Pelvic and	1982/1/12
LGE0045	No		Yes	No	Yes	Progestin	12				Pelvic cavity	1967/6/2
LGE0047	No		Yes	Yes	No	Progestin	6		PEI	4	Lung	1973/4/26
LGE0051	No		No	Yes	No				PI	4	Vaginal stump	1966/1/15
LGE0053	No		Yes	No	No	Progestin	14				Vaginal stump	1953/5/17
LGE0054	Yes	No	Yes	No	No	Aromatase inhibitor	2				Pelvic and	1972/6/5
LGE0055	Yes	No	Yes	No	No	Aromatase inhibitor	3				Rectum	1964/12/8
LGE0057	No		No	No	No						Pelvic cavity	1957/10/4
LGE0058	No		No	No	No						Vaginal stump	1960/2/9
LGE0059	No		No	No	No						Pelvic cavity	1966/10/27
LGE0061	No		No	No	No						Lung	1958/9/15
LGE0062	Yes	No	No	No	No						Vaginal stump	1968/6/22
LGE0063	No		Yes	No	No	GnRHa	6					1982/3/4
LGE0064	No		Yes	No	No	GnRHa/Aromatase inhibitor	5					1984/12/8
LGE0065	No		Yes	No	No	Progestin	12					1989/10/13
LGE0066	No		Yes	No	No	Progestin	6					1998/1/18
LGE0067	No		No	No	No							1978/3/22
LGE0068	No		Yes	No	Yes	Progestin	36					1963/8/12
LGE0069	No		Yes	Yes	No	Progestin	3		PEI	3		1985/7/7
LGE0070	No		No	Yes	No				PEI	5		1951/6/10
LGE0071	No		Yes	No	Yes	Progestin	6					1981/11/2
LGE0072	No		No	No	No							1970/8/31
LGE0073	No		Yes	No	No	GnRHa/LNG-IUS	55					1982/9/4
LGE0074	No		Yes	No	No	GnRHa/LNG-IUS	90					1997/7/1
LGE0075	No		Yes	No	No	LNG-IUS	24					1981/2/16

LGE0076	No		Yes	No	No	Progestin	6			1992/6/19
LGE0077	No		No	No	No					1994/11/9
LGE0078	No		No	No	No					1978/1/23
LGE0079	No		Yes	No	No	GnRHa	2			1985/5/4
LGE0080	No		Yes	No	No	GnRHa	4			1978/3/20
LGE0081	No		Yes	No	No	GnRHa/Progestin	12			1984/5/6
LGE0082	No		Yes	No	No	Progestin	6			1986/8/30
LGE0083	No		Yes	No	No	Progestin	6			1982/8/10
LGE0084	No		Yes	No	No	Progestin	6			1977/12/7
LGE0085	No		Yes	No	No	Progestin	12			1991/12/3
LGE0086	No		Yes	No	No	Progestin	4			1981/5/12
LGE0087	No		Yes	No	No	Progestin	8			1994/12/10
LGE0088	No		No	No	No					1984/10/19
LGE0089	No		No	No	No					1973/11/1
LGE0090	No		No	No	No					1974/7/5
LGE0091	No		No	No	No					1984/7/20
LGE0092	Yes	No	No	Yes	Yes			PI/PA	6	1979/1/5
LGE0093	Yes	No	Yes	No	Yes	GnRHa	3			1965/1/30
LGE0094	No		Yes	No	Yes	GnRHa/Progestin/Aromatase inhibitor	26			1968/12/22
LGE0095	No		Yes	No	Yes	GnRHa/Aromatase inhibitor	4			1975/5/2
LGE0096	No		Yes	No	Yes	Progestin	12			1969/11/22
LGE0097	No		Yes	No	Yes	Progestin	12			1967/4/17
LGE0098	Yes	No	Yes	No	Yes	Progestin	16			1974/5/6
LGE0099	No		Yes	No	Yes	Progestin	14			1969/12/4
LGE0101	Yes	No	No	No	Yes					1964/11/25
LGE0102	No		No	No	Yes					1954/5/6
LGE0103	Yes	No	No	No	Yes					1971/12/30
LGE0104	No		No	No	Yes					1981/10/3
LGE0105	Yes	No	No	No	Yes					1965/5/4
LGE0106	No		Yes	Yes	No	Progestin	12	PEI	4	1975/12/3
LGE0107	No		Yes	Yes	No	Aromatase inhibitor	12	PEI	6	1968/4/6
LGE0108	No		No	Yes	No			PEI	1	1976/9/30
LGE0109	Yes	No	No	Yes	No			PEI	3	1976/12/26
LGE0110	No		No	Yes	No			PEI	3	1984/7/21
LGE0111	No		Yes	No	No	GnRHa	4			1996/2/6
LGE0112	No		Yes	No	No	GnRHa	3			1984/12/27
LGE0113	No		Yes	No	No	GnRHa/Aromatase inhibitor	6			1968/2/21
LGE0114	No		Yes	No	No	Progestin	16			1975/12/20
LGE0115	No		Yes	No	No	Progestin	12			1973/7/20
LGE0116	No		Yes	No	No	Progestin	18			1981/10/17
LGE0117	No		Yes	No	No	Progestin	12			1966/10/10
LGE0118	No		Yes	No	No	Progestin	6			1980/10/23
LGE0119	No		Yes	No	No	Progestin	6			1971/7/11
LGE0120	No		Yes	No	No	Progestin	12			1963/10/18
LGE0121	No		Yes	No	No	Progestin	9			1971/10/12

LGE0122	No		Yes	No	No	Progestin	9			1975/3/4
LGE0123	No		Yes	No	No	Progestin	12			1990/10/11
LGE0124	No		Yes	No	No	Progestin	6			1960/2/6
LGE0125	No		Yes	No	No	Progestin	14			1976/12/27
LGE0126	No		Yes	No	No	Progestin	19			1971/5/18
LGE0127	No		Yes	No	No	Progestin	12			1973/12/30
LGE0128	No		Yes	No	No	Progestin	12			1981/12/2
LGE0129	Yes	No	Yes	No	No	Aromatase inhibitor	31			1973/9/3
LGE0130	No		Yes	No	No	Aromatase inhibitor	6			1952/9/7
LGE0131	No		Yes	No	No	Aromatase inhibitor	6			1963/11/8
LGE0132	No		Yes	No	No	Aromatase inhibitor	6			1966/10/26
LGE0133	No		Yes	No	No	Aromatase inhibitor	17			1972/3/29
LGE0134	Yes	No	No	No	No					1971/5/17
LGE0135	No		No	No	No					1969/9/25
LGE0136	No		No	No	No					1967/3/6
LGE0137	No		No	No	No					1963/4/25
LGE0138	No		No	No	No					1965/5/20
LGE0139	No		No	No	No					1986/6/10
LGE0140	No		No	No	No					1972/8/30
LGE0141	No		No	No	No					1969/8/7
LGE0142	No		No	No	No					1975/6/5
LGE0143	No		No	No	No			PEI	3	1978/2/5
LGE0144	No		No	No	No					1988/8/22
LGE0145	No		No	No	No					1975/10/9
LGE0146	Yes	No	No	No	No					1971/6/1
LGE0147	No		No	No	No					1969/1/26
LGE0148	Yes	No	No	No	No					1978/4/27
LGE0149	No		No	No	No					1982/12/22
LGE0150	No		No	No	No					1974/7/6
LGE0151	No		No	No	No					1973/3/13
LGE0152	No		No	No	No					1965/8/10
LGE0153	No		No	No	No					1969/9/16
LGE0154	No		No	No	No					1970/8/14
LGE0155	No		No	No	No					1986/3/1
LGE0156	No		No	No	No					1963/1/26
LGE0157	No		No	No	No					1982/7/7
LGE0158	No		No	No	No					1973/6/9
LGE0159	No		No	No	No					1977/4/17
LGE0160	No		No	No	No					1972/1/17
LGE0161	No		No	No	No					1962/6/15
LGE0162	No		No	No	No					1962/4/24
LGE0163	No		No	No	No					1965/11/27
LGE0164	No		No	No	No					1970/5/27
LGE0165	No		No	No	No					1971/2/3
LGE0166	No		No	No	No					1981/7/14

LGE0167	No		No	No	No	1966/10/17
LGE0168	No		No	No	No	1970/12/2
LGE0169	No		No	No	No	1970/11/16
LGE0170	No		No	No	No	1964/12/22
LGE0171	Yes	No	No	No	No	1972/1/28
LGE0172	No		No	No	No	1968/8/8
LGE0173	No		No	No	No	1950/10/27
