# Supplementary material for: Prognosis of uterine and extrauterine low-grade endometrial stromal sarcoma: an observational cohort study
Source: Int J Surg. 2024 Feb 7;110(4):1919–28. doi: 10.1097/JS9.0000000000001146 (PMC11020013; doi:10.1097/JS9.0000000000001146)
Supplement: Supplementary file 2 [file js9-110-1919-s002.pdf]

No	Extrauterine LGESS sites	LVSI	ER(+)	PR(+)	MI>5	Definite mitotic	Repeated recurrence or progression	Death	PFI (months)	OS (months)
LGE0001		No	Yes	Yes	No	3-5				
LGE0002		No	Yes	No	No	<1				
LGE0003			Yes	Yes	No	3-5	Yes	No	57	118
LGE0004		No	Yes	Yes	No	3-5	Yes	No	5	59
LGE0005			Yes	Yes	No	3-5	Yes	No	18	101
LGE0006		No	Yes	Yes	No	1-3	No	No	16	23
LGE0007		No	Yes	Yes	Yes	6-10				
LGE0008	Ovarian		Yes	Yes	No	3-5	Yes	Yes	8	21
LGE0009		No	Yes	Yes	No	1-3				
LGE0010	Vaginal		Yes	Yes	Yes	>10				
LGE0011	Vaginal	No	Yes	Yes	No	1-3	No	No	40	44
LGE0012	Vaginal	No	Yes	Yes	No	3-5				
LGE0013	Ovarian		Yes	Yes	No	1-3				
LGE0014	Ovarian		Yes	Yes	No	1-3	Yes	No	39	229
LGE0016	Ovarian		Yes	Yes	Yes	>10	Yes	Yes	19	42
LGE0017	Unknown	No	Yes	Yes	Yes	>10	Yes	No	61	23
LGE0018	Vaginal		No	Yes	Yes	>10	Yes	Yes	121	134
LGE0019		No	Yes	Yes	Yes	6-10				
LGE0020		No	Yes	Yes	No	1-3	No	No	32	44
LGE0021		Yes	No	Yes	Yes	>10	Yes	No	3	61
LGE0022		No	Yes	Yes	No	1-3				
LGE0023		No	Yes	No	No	3-5	Yes	No	8	8
LGE0024			Yes	Yes	No	3-5	Yes	No	8	24
LGE0025			Yes	Yes	No	3-5	Yes	No	101	182
LGE0026		No	Yes	Yes	No	3-5	No	No	39	44
LGE0027			Yes	Yes	No	3-5	Yes	No	2	349
LGE0028			No	Yes	No	3-5	Yes	No	37	78
LGE0029			Yes	Yes	No	3-5	Yes	No	46	63
LGE0030		No	Yes	Yes	No	3-5	No	No	58	58
LGE0031			Yes	Yes	No	3-5	No	No	93	99
LGE0032		Yes	Yes	Yes	No	1-3	No	No	3	3
LGE0033			No	Yes	No	3-5	Yes	No	70	118
LGE0034			Yes	Yes	No	1-3	No	No	80	84
LGE0035		No	No	Yes	No	3-5				
LGE0037			Yes	Yes	No	1-3	Yes	Yes	18	37
LGE0038			Yes	Yes	No	<1	Yes	Yes	91	115
LGE0039		Yes	Yes	No	No	3-5	No	No	37	44
LGE0040		Yes	Yes	Yes	No	3-5	No	No	15	27
LGE0041		No	Yes	Yes	Yes	6-10	Yes	Yes	1	24
LGE0042			Yes	Yes	No	1-3	Yes	No	16	45
LGE0043			Yes	Yes	No	1-3	Yes	No	38	101
LGE0044		Yes	Yes	Yes	No	1-3	Yes	No	10	26
LGE0045		Yes	Yes	Yes	Yes	>10	Yes	No	25	38

LGE0046			Yes	No	No	3-5	Yes	Yes	5	14
LGE0048	Ovarian		Yes	Yes	No	3-5	Yes	Yes	16	36
LGE0049			Yes	Yes	No	<1	Yes	Yes	12	67
LGE0050			Yes	Yes	No	1-3	No	No	60	65
LGE0051		No	Yes	Yes	Yes	6-10	Yes	Yes	3	12
LGE0052			Yes	Yes	No	1-3	Yes	Yes	3	39
LGE0053		No	Yes	Yes	No	1-3	No	No	3	3
LGE0054		No	Yes	No	No	<1	Yes	No	2	29
LGE0055		No	Yes	Yes	No	3-5	No	No	62	62
LGE0058		No	Yes	No	No	3-5				
LGE0059		No	Yes	Yes	Yes	>10				
LGE0061		No	Yes	Yes	No	3-5	Yes	Yes	2	14
LGE0062		No	Yes	Yes	No	<1				
	**Previous DFS (months)**	**Fertility sparing**	**Ovarian preservat ion**	**Repeated fertility sparing**	**Repeated ovarian preservat ion**	**Extrauter ine LGESS**	**Ages at diagnosis (years)**	**Ages at recurr ence (years**	**Recurrence within pelvic cavity**	**Recurrent sites within pelvic cavity**
LGE0001		No	No	No	No	No	26	29	Yes	Uterus
LGE0002		No	No	No	No	No	28	34	Yes	Uterus
LGE0003	49	No	Yes	No	Yes	No	29	33	Yes	Pelvic cavity
LGE0004	19	Yes	Yes	Yes	Yes	No	25	26	Yes	Retroperiton eal lymph
LGE0005	29	Yes	Yes	Yes	Yes	No	31	34	No	
LGE0006	23	No	No	No	No	No	29	30	Yes	Pelvic cavity
LGE0007		No	No	No	No	No	37	37	Yes	Pelvic cavity
LGE0008	39	Yes	Yes			Yes	33	36	Yes	Pelvic cavity
LGE0009		No	No	No	No	No	33	35	Yes	Pelvic cavity
LGE0010		No	No	No	No	Yes	34	42	Yes	Uterus, ovaries
LGE0011	34	No	No	No	No	Yes	36	38	Yes	Vagina
LGE0012		Yes	Yes	Yes	Yes	Yes	48	52	Yes	Vagina
LGE0013		No	No		No	Yes	43	45	Yes	Ovaries
LGE0014	96	No	No			Yes	46	54	Yes	Pelvic cavity
LGE0016	133	No	No			Yes	45	56	Yes	Pelvic cavity
LGE0017	78	No	No		No	Yes	50	56	Yes	Pelvic and peritoneal
LGE0018	122	No	No			Yes	43	53	Yes	Pelvic cavity
LGE0019		No	No	No	No	No	35	36	Yes	Uterus, ovaries
LGE0020	17	No	No	No	No	No	39	40	Yes	Pelvic cavity
LGE0021	6	Yes	Yes	Yes	Yes	No	16	17	Yes	Pelvic cavity
LGE0022		No	No	No	No	No	35	37	Yes	Uterus
LGE0023	58	Yes	Yes	Yes	Yes	No	20	25	Yes	Uterus
LGE0024	146	No	No			No	44	56	Yes	Pelvic cavity
LGE0025	46	No	No		No	No	31	35	Yes	Vaginal stump
LGE0026	43	No	No		No	No	52	56	Yes	Pelvic cavity
LGE0027	5	No	Yes			No	27	27	Yes	Pelvic cavity
LGE0028	33	No	Yes			No	18	21	Yes	Pelvic cavity
LGE0029	83	No	No		No	No	24	31	Yes	Pelvic cavity

LGE0030	29	No	No		No	No	35	37	Yes	Pelvic cavity
LGE0031	40	No	No		No	No	21	24	Yes	Rectum
LGE0032	24	No	No		No	No	28	30	Yes	Vaginal stump
LGE0033	35	No	No		No	No	37	40	Yes	Vaginal stump
LGE0034	167	No	No		No	No	57	70	Yes	Pelvic cavity, retroperitone
LGE0035		No	No		No	No	44	45	Yes	Vaginal stump
LGE0037	61	No	No		No	No	44	49	Yes	Retroperiton eal lymph
LGE0038	143	No	No		No	No	45	57	Yes	Vaginal stump
LGE0039	14	No	No		No	No	50	51	Yes	Vaginal stump
LGE0040	5	No	No		No	No	46	47	Yes	Vaginal stump
LGE0041	6	No	No		No	No	36	37	Yes	Pelvic cavity
LGE0042	182	No	No			No	26	41	Yes	Rectum
LGE0043	88	No	No			No	47	54	Yes	Pelvic cavity
LGE0044	19	No	No			No	35	36	Yes	Pelvic cavity
LGE0045	62	No	No			No	45	50	Yes	Pelvic cavity
LGE0046	147	No	No			No	43	55	Yes	Pelvic cavity
LGE0048	18	No	No			Yes	60	61	Yes	Pelvic cavity
LGE0049	44	No	No			No	26	29	Yes	Abdominal wall
LGE0050	188	No	No			No	53	69	Yes	Vaginal stump
LGE0051	6	No	No			No	52	52	Yes	Vaginal stump
LGE0052	6	No	No			No	52	53	Yes	Pelvic cavity
LGE0053	27	No	No			No	65	67	Yes	Vaginal stump
LGE0054	9	No	No			No	45	46	Yes	Pelvic cavity
LGE0055	6	No	No			No	50	51	Yes	Rectum
LGE0058		No	No			No	55	58	Yes	Vaginal stump, pelvic
LGE0059		No	No			No	49	51	Yes	Pelvic cavity
LGE0061	11	No	No			No	58	59	No	
LGE0062		No	No			No	48	48	Yes	Vaginal stump

	Recurrence beyond pelvic cavity	Recurren t sites beyond	Symptom s at recurrenc	Symptom s	Dignostic methods for first	Definite diagnosti c	Repeated surgeries	Resid ual disea	Residual sites	Chemothera py for first recurrence
LGE0001	Yes	Lung	No		Imaging	Imaging: MRI	Yes	No		Yes
LGE0002	Yes	Lung	Yes	Vaginal bleeding	Imaging	Imaging: MRI	Yes	No		No
LGE0003	Yes	Abdomina l wall	Yes	Vaginal bleeding	Imaging	Imaging: sonograp	Yes	No		Yes
LGE0004	No		No		Exploratio n during	Exploratio n during	Yes	No		No
LGE0005	Yes	Abdomina l wall,	Yes	Abdomina l mass	Imaging	Imaging: sonograp	Yes	No		No
LGE0006	Yes	Liver, omentum	No		Imaging	Imaging: MRI	Yes	No		No
LGE0007	No		No		Imaging	Imaging: sonograp	Yes	No		No
LGE0008	No		No		Imaging	Imaging: sonograp	No			No
LGE0009	No		Yes	Abdomina l mass	Imaging	Imaging: sonograp	Yes	No		No
LGE0010	Yes	Perikidney	No		Imaging	Imaging: MRI	Yes	No		No
LGE0011	No		Yes	Vaginal mass	Physical examinati	Physical examinati	Yes	No		No
LGE0012	No		Yes	Vaginal mass	Physical examinati	Physical examinati	Yes	No		No
LGE0013	Yes	Groin lymph	Yes	Abdomina l pain	Imaging	Imaging: sonograp	Yes	No		No
LGE0014	No		Yes	Abdomina l mass	Imaging	Imaging: sonograp	Yes	No		Yes
LGE0016	No		No		Imaging	Imaging: MRI	No			Yes

LGE0017	Yes	Omentum	Yes	Abdomina l pain and	Imaging	Imaging: CT	Yes	No		No
LGE0018	No		No		Physical examinati	Physical examinati	Yes	No		No
LGE0019	Yes	Liver	No		Imaging	Imaging: sonograp	Yes	No		No
LGE0020	Yes	Peritoneal cavity	No		Imaging	Imaging: PET-CT	Yes	No		No
LGE0021	No		No		Imaging	Imaging: sonograp	Yes	No		No
LGE0022	No		No		Imaging	Imaging: sonograp	Yes	No		No
LGE0023	No		No		Imaging	Imaging: sonograp	Yes	No		No
LGE0024	No		Yes	Backache	Imaging	Imaging: CT	Yes	Yes	Pelvic cavity	No
LGE0025	No		No		Physical examinati	Physical examinati	Yes	No		Yes
LGE0026	No		No		Imaging	Imaging: sonograp	Yes	No		No
LGE0027	No		Yes	Abdomina l pain	Physical examinati	Physical examinati	No			No
LGE0028	Yes	Lung	Yes	Abdomina l pain	Imaging	Imaging: PET-CT	No			Yes
LGE0029	Yes	Lung	No		Imaging	Imaging: sonograp	Yes	No		Yes
LGE0030	Yes	Abdomina l wall	No		Imaging	Imaging: sonograp	Yes	No		No
LGE0031	Yes	Lung	Yes	Abdomina l pain and	Imaging	Imaging: PET-CT	Yes	No		Yes
LGE0032	No		Yes	Abdomina l pain	Imaging	Imaging: CT	Yes	No		No
LGE0033	No		No		Physical examinati	Physical examinati	Yes	No		Yes
LGE0034	Yes	Lung	No		Imaging	Imaging: CT	Yes	No		No
LGE0035	Yes	Lung, bladder	Yes	Vaginal bleeding	Physical examinati	Physical examinati	Yes	No		No
LGE0037	No		Yes	Abdomina l mass	Physical examinati	Physical examinati	Yes	No		No
LGE0038	No		Yes	Vaginal bleeding	Physical examinati	Physical examinati	Yes	No		No
LGE0039	Yes	Bladder	Yes	Backache	Imaging	Imaging: CT	Yes	No		No
LGE0040	No		Yes	Backache	Imaging	Imaging: sonograp	Yes	No		Yes
LGE0041	No		No		Imaging	Imaging: CT	Yes	No		No
LGE0042	Yes	Omentum	Yes	Abdomina l pain	Imaging	Imaging: CT	Yes	No		Yes
LGE0043	No		No		Imaging	Imaging: sonograp	Yes	No		No
LGE0044	Yes	Peritoneal cavity	No		Imaging	Imaging: CT	Yes	No		Yes
LGE0045	No		No		Imaging	Imaging: sonograp	Yes	No		No
LGE0046	Yes	Peritoneal cavity	No		Imaging	Imaging: CT	Yes	Yes	Intestine	Yes
LGE0048	No		Yes	Ileus	Imaging	Imaging: sonograp	Yes	No		Yes
LGE0049	Yes	Abdomina l wall	Yes	Abdomina l mass	Physical examinati	Physical examinati	Yes	No		No
LGE0050	Yes	Peritoneal cavity	No		Imaging	Imaging: sonograp	Yes	No		No
LGE0051	No		No		Imaging	Imaging: CT	No			No
LGE0052	No		No		Imaging	Imaging: CT	Yes	No		No
LGE0053	No		No		Imaging	Imaging: sonograp	Yes	No		Yes
LGE0054	Yes	Lung, peritoneal	No		Imaging	Imaging: PET-CT	Yes	No		Yes
LGE0055	No		No		Imaging	Imaging: MRI	Yes	No		No
LGE0058	No		No		Imaging	Imaging: sonograp	Yes	Yes	Right pelvic wall	No
LGE0059	No		No		Imaging	Imaging: sonograp	Yes	No		No
LGE0061	Yes	Lung	Yes	Fatigue	Imaging	Imaging: CT	No			Yes
LGE0062	No		No		Physical examinati	Physical examinati	Yes	No		No
	**Chemotherapy regimens**	**Chemotherapy courses**	**Radiotherapy for first recurrenc**	**Hormone therapy for first recurrenc**	**Hormone regimens**	**Hormone therapy periods (months)**	**Repeated recurrenc or progression**	**Previous history of**	**Recurrent sites**	**Date of last treatment for first recurrence**

LGE0001	Nedaplatin/Epirubicin/Ifosfamide	1	No	Yes	Progestin/GnRHa	10		No		2016/4/21
LGE0002			No	Yes	Progestin	12		No		2018/3/28
LGE0003	Cisplatin	2	No	No			2016-9-22	No	Pelvic and peritoneal	2012/1/9
LGE0004			No	No			2016/4/1	No	Uterus	2015/11/6
LGE0005			No	Yes	Progestin	6	2013/12/1	No	Abdominal wall, uterus	2012/6/13
LGE0006			No	Yes	Progestin	6	2020/9/1	No		2019/5/5
LGE0007			No	No				No		2015/4/30
LGE0008			No	Yes	GnRHa	1	2016/10/1	No	Pelvic cavity	2016/2/1
LGE0009			No	Yes	Progestin/GnRHa/ar	33		No		2014/9/12
LGE0010			Yes	No				No		2015/2/1
LGE0011			No	No			2020/9/1	No		2017/5/23
LGE0012			Yes	No				No		2016/12/1
LGE0013			No	Yes	Progestin	9		No		2012/6/7
LGE0014	PEB	1	No	No			2005/2/1	No	Pelvic cavity	2001/12/1
LGE0016	Liposome doxorubicin/apatinib	1	No	Yes	Progestin	12	2019/8/1	No	Pelvic cavity, lung	2018/1/12
LGE0017			No	Yes	Progestin	30	2018/10/1	No	Liver, retroperitoneal	2013/9/24
LGE0018			Yes	No			2017/12/7	No	Pelvic cavity, colorectum	2008/1/1
LGE0019			No	No				No		2018/9/1
LGE0020			No	No			2020/9/1	No		2018/1/4
LGE0021			No	No			2015/11/19	Yes	Ovary, uterus, pelvic	2015/9/1
LGE0022			No	No				No		2015/12/25
LGE0023			No	Yes	GnRHa	6	2015/12/16	No	Retroperitoneal lymph	2015/4/9
LGE0024			Yes	Yes	Progestin	2	2019/11/1	No	Pelvic cavity	2019/3/4
LGE0025	PEI	3	No	No			2014-3-7	No	Pelvic cavity	2005/12/1
LGE0026			Yes	Yes	Progestin	41	2020/9/1	No		2017/7/3
LGE0027			Yes	No			1992/1/1	No	Pelvic cavity	1991/11/16
LGE0028	PEI	6	No	Yes	Progestin	2	2017/6/1	Yes	Pelvic cavity, lung	2014/5/7
LGE0029	PEI	2	Yes	Yes	Progestin/GnRHa	40	2020/9/1	Yes	Pelvic cavity	2016/12/1
LGE0030			No	No			2020/9/1	Yes		2015/12/10
LGE0031	PEI	7	No	Yes	Progestin	24	2020/9/1	No		2013/1/26
LGE0032			No	Yes	Progestin	3	2020/9/1	No		2020/6/12
LGE0033	PIA	5	No	No			2017/5/1	No	Pelvic cavity, lung	2011/8/1
LGE0034			Yes	No			2020/9/1	No		2014/2/1
LGE0035			Yes	No				No		2014/11/1
LGE0037			No	No			2014/2/1	No	Pelvic and peritoneal	2012/8/2
LGE0038			Yes	No			2014/9/1	No	Pelvic and peritoneal	2007/3/15
LGE0039			Yes	Yes	Progestin	39	2020/9/1	No		2017/8/30
LGE0040	Liposome doxorubicin	6	No	Yes	Progestin	13	2020/9/1	No		2019/6/6
LGE0041			No	No			2016/3/1	Yes	Pelvic and peritoneal	2016/1/26
LGE0042	PEI	3	No	Yes	Progestin	11	2020/5/13	Yes	Intestine, liver	2019-2-2
LGE0043			No	Yes	Progestin	12	2015/10/1	No	Pelvic cavity	2012/8/15
LGE0044	PE	4	No	No			2019/9/19	No	Pelvic and peritoneal	2018/12/6
LGE0045			No	Yes	Progestin	3	2019/9/11	No	Pelvic and peritoneal	2017/8/25
LGE0046	TC	6	No	No			2014-3-20	Yes	Pelvic cavity, liver	2013/10/14
LGE0048	TC/Epirubicin	7	No	Yes	Progestin	15	2014-2-25	Yes	Pelvic cavity	2012/11/13

LGE0049			No	Yes	Progestin	6	2013/1/1	No	Bladder	2012/1/1
LGE0050			Yes	No			2020/9/1	No		2015/10/1
LGE0051			Yes	No			2018/6/1	No	Pelvic cavity	2018/3/1
LGE0052			Yes	No			2011-1-17	No	Liver	2010/10/21
LGE0053	Epirubicin	1	Yes	Yes	Progestin	3	2020/9/1	No		2020/6/18
LGE0054	PEI/PI*3/PD-1*2	6	No	Yes	Progestin	3	2018/9/6	Yes	Pelvic cavity, lung	2018/7/21
LGE0055			No	Yes	Progestin	12	2020/9/1	Yes		2015/8/11
LGE0058			Yes	No				No		2018/1/1
LGE0059			Yes	No				No		2018/1/1
LGE0061	Epirubicin	2	Yes	No			2017/12/1	No	Lung	2017/9/27
LGE0062			No	No				Yes		2016/9/19
